# Molecular characterization of accripin11, a soluble shell protein with an acidic C‐terminus, identified in the prismatic layer of the Mediterranean fan mussel *Pinna nobilis* (Bivalvia, Pteriomorphia)

**DOI:** 10.1002/2211-5463.13497

**Published:** 2022-12-03

**Authors:** Benazir Khurshid, Daniel J. Jackson, Sylvain Engilberge, Sébastien Motreuil, Cédric Broussard, Jérôme Thomas, Françoise Immel, Matthew J. Harrington, Peter B. Crowley, Daniel Vielzeuf, Jonathan Perrin, Frédéric Marin

**Affiliations:** ^1^ Laboratoire Biogéosciences, UMR CNRS‐EPHE 6282 Université de Bourgogne – Franche‐Comté Dijon France; ^2^ Synchrotron SOLEIL Beamline ANATOMIX Gif‐sur‐Yvette France; ^3^ Department of Geobiology Georg‐August University of Göttingen Germany; ^4^ Structural Biology Group European Synchrotron Radiation Facility Grenoble France; ^5^ Plateforme Protéomique 3P5 Université Paris Descartes France; ^6^ Chrono‐Environnement, UMR 6249 CNRS Université de Bourgogne Franche‐Comté Besançon France; ^7^ Department of Chemistry McGill University Montreal Canada; ^8^ School of Biological and Chemical Sciences National University of Ireland Galway Ireland; ^9^ Laboratoire CINaM‐CNRS Université d'Aix‐Marseille France

**Keywords:** Alphafold2, biomineral, calcitic prisms, mollusk shell, protein

## Abstract

We have identified a novel shell protein, accripin11, as a major soluble component of the calcitic prisms of the fan mussel *Pinna nobilis*. Initially retrieved from a cDNA library, its full sequence is confirmed here by transcriptomic and proteomic approaches. The sequence of the mature protein is 103 residues with a theoretical molecular weight of 11 kDa and is moderately acidic (pI 6.74) except for its C‐terminus which is highly enriched in aspartic acid. The protein exhibits a peculiar cysteine pattern in its central domain. The full sequence shares similarity with six other uncharacterized molluscan shell proteins from the orders Ostreida, Pteriida and Mytilida, all of which are pteriomorphids and produce a phylogenetically restricted pattern of nacro‐prismatic shell microstructures. This suggests that accripin11 is a member of a family of clade‐specific shell proteins. A 3D model of accripin11 was predicted with AlphaFold2, indicating that it possesses three short alpha helices and a disordered C‐terminus. Recombinant accripin11 was tested *in vitro* for its ability to influence the crystallization of CaCO_3_, while a polyclonal antibody was able to locate accripin11 to prismatic extracts, particularly in the acetic acid‐soluble matrix. The putative functions of accripin11 are further discussed in relation to shell biomineralization.

## Graphical abstract

Accripin11, a novel 11 kDa protein exhibiting a peculiar cysteine pattern and an acidic disordered C‐terminus, was identified in the outer shell layer of the Mediterranean fan mussel, *Pinna nobilis*. It is a member of a protein family restricted to Pteriomorphia bivalves. Accripin11 interacts with the precipitation of CaCO_3_ and is presumably involved in the biomineralization of the shell prismatic microstructure.

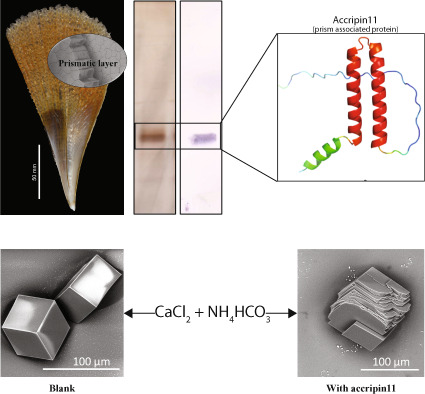

**Abbreviations**
AA: amino acid
AIM: acid insoluble matrix
ASM: acid soluble matrix
CA: carbonic anhydrase
C‐ter: C‐terminus
Cys: cysteine
ELISA: enzyme‐linked immunosorbent assay
GAR‐AP: goat anti‐rabbit alkaline phosphatase conjugate
LCDs: low‐complexity domains
LS: Laemmli soluble
LS‐AIM: Laemmli‐soluble‐acid insoluble matrix
ORF: open reading frame
pI: isoelectric point
RLCDs: repetitive low‐complexity domains

Mollusk shells are natural composite materials that display exceptional mechanical properties, despite being synthesized at ambient temperatures, pressures and biological pH values [1]. These properties emerge from the complex and hierarchical arrangement of crystalline units in well‐defined microstructures exemplified by nacre, prisms and crossed‐lamellar. Their construction is controlled by extracellular organic macromolecules, many of which become occluded in the mature mineral phase. While many mollusk shells are mostly made of two to three layers of calcium carbonate (calcite or aragonite, or the association of the two polymorphs), they also contain a minute amount (usually < 1%) of proteins, glycoproteins, polysaccharides, pigments and lipids [2] that drastically modify the properties of the mature biomineral.

Of the array of occluded macromolecules, proteins have long been the most studied: 141 years separate the first “modern” chemical analysis of a shell performed by Frémy (which led to the term “conchiolin” [3]) from the publication of the first full sequence of a shell protein, nacrein, which is associated with the nacreous layer of the Japanese pearl oyster [4]. In between, proteins extracted in bulk were the focus of numerous biochemical characterizations (summarized in [5, 6, 7]). Most of these studies have focused on the nacre of diverse species and have disregarded other common types of shell microstructures such as the prismatic layer [8]. On account of its distinctive microstructure, comprising flat aragonitic tablets arranged to hinder crack propagation, nacre possesses a fracture toughness of at least a thousand times higher than that required for fragmenting geological aragonites [9]. Consequently, the knowledge of a limited number of nacre proteins generated an overly optimistic perspective to mimic these properties *in vitro* [10, 11, 12]. Today, with high‐throughput screening techniques, that is, transcriptomics combined with proteomics, it is clear that the deposition of nacre, and likely all types of shell microstructures, requires a large set of proteins of extraordinarily diverse functions [8]. Some of these functions include calcium binding, enzymatic activities, modification of 3D frameworks (e.g., chitin modification), inhibition of protease activity, antibacterial activity and signaling functions. Note that relatively few functions of shell‐forming proteins have been verified by *in vitro* experiments.

Many proteins are occluded into the shell during the shell‐forming process, constituting the shell matrix (the “shellome”) while others – more elusive – are supposed to remain at the interface between the calcifying epithelium and the mineralization front, before being degraded or recycled. Both occluded and non‐occluded proteins are likely to play important roles in mineral deposition, but this aspect, evidenced for the first time 15 years ago [13], remains largely unexplored [14].

The growing list of proteins that are identified as true shell components comprises several members exhibiting functional domains similar to those of proteins found in non‐mineralizing systems. For example, nacrein, referred to above, contains a carbonic anhydrase (CA) domain and, from experimental testing, possesses CA activity [15]. Tyrosinases have been identified and may function in cross‐linking, pigmentation and defense mechanisms [16, 17]. Proteins with a typical extracellular matrix signature, such as Von Willebrand factor type A domain, also belong to this category [18] as well as proteins with protease inhibitor domains [19, 20].

Conversely, the majority of shell‐associated proteins share no sequence similarity with proteins in relatively highly studied non‐calcifying systems. This is typically the case of proteins with primary structures dominated by low‐complexity domains (LCDs) or repetitive low‐complexity domains (RLCDs), also called “compositionally biased regions.” Almost none of the LCD/RLCD‐containing proteins have been assigned unequivocal molecular functions in biomineralization, while their abundance in calcified tissues as well as their diversity remains enigmatic [8]. Some exceptions, such as aspartic acid‐/glutamic acid‐rich domain containing proteins, were hypothesized to interact with calcium ions or with calcium carbonate surfaces long before the actual discovery of their primary structures [21, 22]. Among the shell proteins with no homologs in non‐calcifying systems, one also finds members that do not exhibit any LCDs/RLCDs in spite of having biased AA composition. For instance, upsalin [23], found in the nacreous layer of the freshwater mussel *Unio pictorum*, is of that type.

Here we describe accripin11, which belongs to this second category but exhibits a composite primary structure, while 90% of its sequence does not have any homology with known proteins of non‐mineralizing systems and is only slightly “compositionally biased,” its C‐terminus contains a short LCD with an aspartic acid‐rich domain. Accripin11 was identified in the shell of the fan mussel *Pinna nobilis*, a species endemic to the Mediterranean Sea and also its largest bivalve representative. This species is on the verge of extinction due to a pandemic caused by a protozoan parasite [24, 25]. As for many pteriomorphid bivalves, the shell of *P. nobilis* possesses two calcified layers comprising an internal aragonitic nacreous layer and an external calcitic layer made of long prisms, probably the longest monocrystal‐like biominerals in the mollusk world, and a fascinating model *per se* to explore the mechanisms of shell formation.

## Results

### Overall strategy to identify and characterize accripin11

**Fig. 1:**
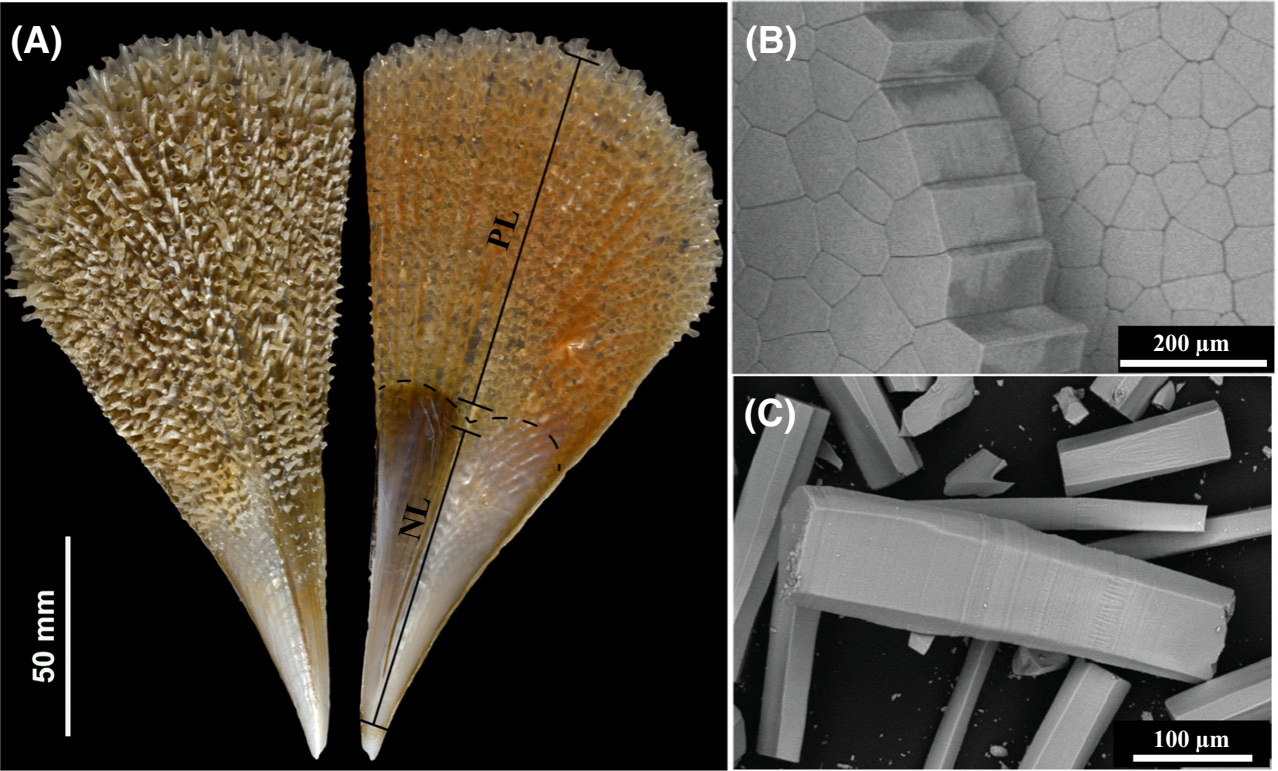
Shell structure of *Pinna nobilis*. (A) External and internal macroscopic view of the shell from a juvenile specimen of approx. age 1.5 years, size 200 mm. The lustrous lower half part is the nacreous layer (NL) while the calcitic prismatic layer (PL) corresponds to the top brownish part of the shell. The two layers are separated by dashed lines. Scale bar: 50 mm. (B) The prismatic layer of the cleaned shell of 2.5‐year‐old specimen observed under SEM in backscattering mode. The prismatic layer is composed of polygonal calcitic prisms that are stacked together by a thin organic sheath. Scale bar: 200 μm. (C) SEM image of isolated prism that is composed of several nano‐stacks; prisms were isolated with sodium hypochlorite. Scale bar: 100 μm.

Here, we describe a novel shell protein derived from the Mediterranean fan mussel, *P. nobilis* (Fig. 1A). This protein was identified in the external reddish‐brown layer made of long prismatic calcitic crystals that exhibit a polygonal section. The prisms are maintained together by a thin organic honeycomb‐like matrix (Fig. 1B). They exhibit elongation axis orthogonal to the outer shell surface and grow inwards in the direction of the epithelium that generate them. The prisms can be entirely dissociated (Fig. 1C) by degrading the peri‐prismatic sheath with dilute sodium hypochlorite (bleach).

The strategy to identify accripin11 was conducted in two stages separated by a dozen years: initially, its sequence was obtained by antibody screening of a cDNA library but was never published, only mentioned as CSP3 in a review paper on *P. nobilis* [26]. The sequence was obtained from an isolated Lambda‐Zap clone that contained an insert encoding a 363 bp ORF.

The second step corresponds to the work presented here: the sequence was retrieved and confirmed by combining a transcriptome (SRA database, BioProject accession number PRJNA887567 at NCBI) made from the mantle tissue brim, that is supposed to secrete solely the prismatic outer layer, and proteomics performed on shell extracts of the isolated prismatic layer. In the transcriptome of the mantle brim of *P. nobilis*, we identified a transcript (> Pnobilis_R20673250, see Fig. S1), in which the longest open reading frame (121 AA) encodes the protein identified in the clone some years earlier. This protein was retrieved via proteomics on the prism matrix, and its sequence was covered at 93% by 40 peptides (Fig. S2), with several overlaps. For the first time, we obtained a putative 3D structure by using AlphaFold2 program. The protein was overexpressed in a bacterial strain, and the overexpressed recombinant protein was doubly purified by affinity and by preparative electrophoresis before being tested further *in vitro*. We named this protein accripin11, corresponding to the following acronym: Acidic C‐terminus, Cysteine‐rich protein of *Pinna nobilis*.

### Characterization of the primary structure of accripin11

**Fig. 2:**
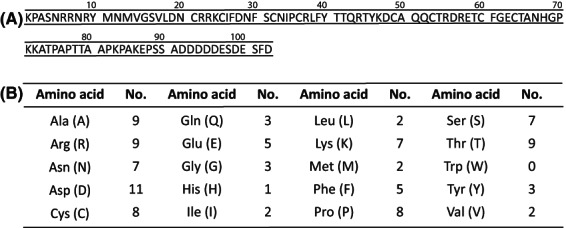
Sequence and composition of accripin11. (A) Amino acid sequence of accripin11, the 11‐kDa protein of the prismatic matrix. (B) Amino acid composition of mature accripin11 (excluding signal peptide), presented as number of residues.

The mature accripin11 is 103 residues long, with a molecular weight of 11.6 kDa and a theoretical pI of 6.7 (Fig. 2A), whereas the full‐length accripin11 is 121 residues. The protein is predicted to be secreted, since a signal‐peptide could be recognized with an unambiguous cleavage site between Ala18 and Lys19 (Fig. S1). Figure 2B shows the overall AA composition of accripin11 after signal peptide cleavage. This composition is slightly biased in comparison to other shell proteins, since the most abundant amino acid is Asp (10.7%), followed by Arg, Ala and Thr (8.7%) then by Cys and Pro (7.8%). The primary structure of mature accripin11 is enriched in small and charged residues as well as proline (8), but depleted in aliphatic residues (only two for Val, Ile, Leu and Met) suggesting that it may be an intrinsically disordered protein. On the other hand, the protein contains Phe (5), Tyr (3) and Cys (8), with the possibility of four disulfide bridges. While accripin11 is overall slightly acidic, there is a distinct bipartite charge distribution. The first 90 residues are enriched in basic residues, including Arg (9) and Lys (7). In contrast, the C‐terminus is exceptionally acidic with seven Asp and two Glu in a 13‐residue stretch.

**Fig. 3:**
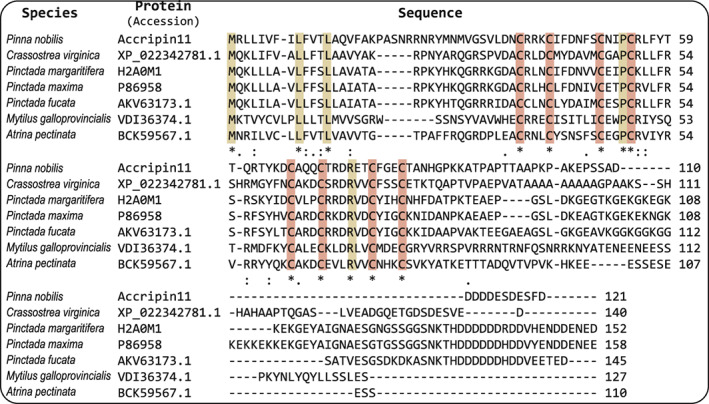
Sequence alignment of accripin11 (including signal peptides) with six low‐molecular‐weight molluscan shell proteins. These proteins were retrieved from pteriomorphid bivalves, including *Pinctada margaritifera*, *Pinctada maxima*, *Pinctada fucata*, *Atrina pectinata* and *Mytilus galloprovincialis*. Amino acid aligned in all seven proteins is shaded in colored boxes. Except accripin11, all proteins are presented with their UniProt accession number.

A standard blast search indicates that accripin11 exhibits sequence similarity only with six low molecular weight unnamed proteins of molluscan origin (Fig. 3) that are all without exception presumably associated with the shell: they include three proteins from pearl oysters of the *Pinctada* genus (Polynesian, i.e., *P. margaritifera*; Australian, i.e., *P. maxima*; Japanese, *P. fucata*), one from the comb pen shell *Atrina pectinata*, one from the edible eastern oyster *Crassostrea virginica* and one from the edible Mediterranean mussel *Mytilus galloprovincialis*. Five of them are uncharacterized shell proteins, while the sixth one (*M. galloprovincialis*) is a hypothetical predicted protein, identified by genome assembly and annotation. The percent identity shared between accripin11 and the other six members is moderate, ranging from 32% to 41% (with signal peptide) and 36% to 48% (without signal peptide). While the N‐termini partly align (at positions 20, 21, 31, 32, 33), all seven proteins align perfectly (with a single gap) in their central domain, in particular the eight cysteine residues at invariant positions, as well as two arginine residues – one flanking downstream the first Cys at position 40, the second between the 6th and 7th Cys residue – and one proline at position 53. Six other positions give an almost perfect match in the central region: Arg55, Leu56, Gln61, Tyr64, Ala68 and Asp74.

The seven C‐termini corresponding (in accripin11) to the 22 AA‐long hydrophobic domain plus the 17 AA‐long Asp‐rich tail match poorly and have different lengths, comprised between 39 (accripin11) and 81 residues (VDI36374.1, *P. maxima*). Interestingly, all seven C‐termini are acidic, but to different degrees: the least acidic is that of *M. galloprovincialis* and the most are from the three *Pinctada* shell proteins. Patterns of note are the poly‐Asp motif of the *Pinctada* species and the poly‐Ala block / Ala‐rich motif flanking the acidic C‐terminus in the *C. virginica* protein (see XP_022342781.1 in Fig. 3).

The perfect alignment of the seven proteins according to their cysteine pattern, the identical motif organization of their primary structure, a short N terminus, the Cys‐rich motif flanked downstream by a hydrophobic domain terminated by a highly acidic tail, in addition to the fact that all seven exhibit similar molecular size, are all of molluscan origin and are all presumably shell proteins, lead us to assign them to a single protein family. The alignment (Fig. 3) indicates that the shell proteins from the three *Pinctada* species exhibit the highest percentage of identity. These three members cluster with the protein from *C. virginica*, then with that of *A. pectinata*, then with accripin11. The hypothetical protein sequence from the mussel *M. galloprovincialis* shares less similarities with the other six members.

We used the Pattinprot tool with the following cysteine pattern: C‐x(3)‐C‐x(6)‐C‐x(3)‐C‐x(12)‐C‐x(3)‐C‐x(6)‐C‐x(3)‐C to identify other proteins exhibiting such a motif (see Fig. S2). We identified four additional proteins: two of them (from mouse and human) are of high molecular weight and contain zinc finger patterns. In both cases, accripin11 aligns with one of the NF‐X1‐type domain located at the C‐terminal part of these proteins. The other proteins with identical Cys pattern comprise a small cysteine and glycine repeat‐containing protein and a keratin‐associated protein, both supposedly involved in cross‐linking with cysteine residues of keratins. However, the overall similarity of accripin11 with these proteins is low, suggesting that, in spite of sharing the same Cys pattern, they belong to completely different protein families.

### 3D model of accripin11 via AlphaFold2

**Fig. 4:**
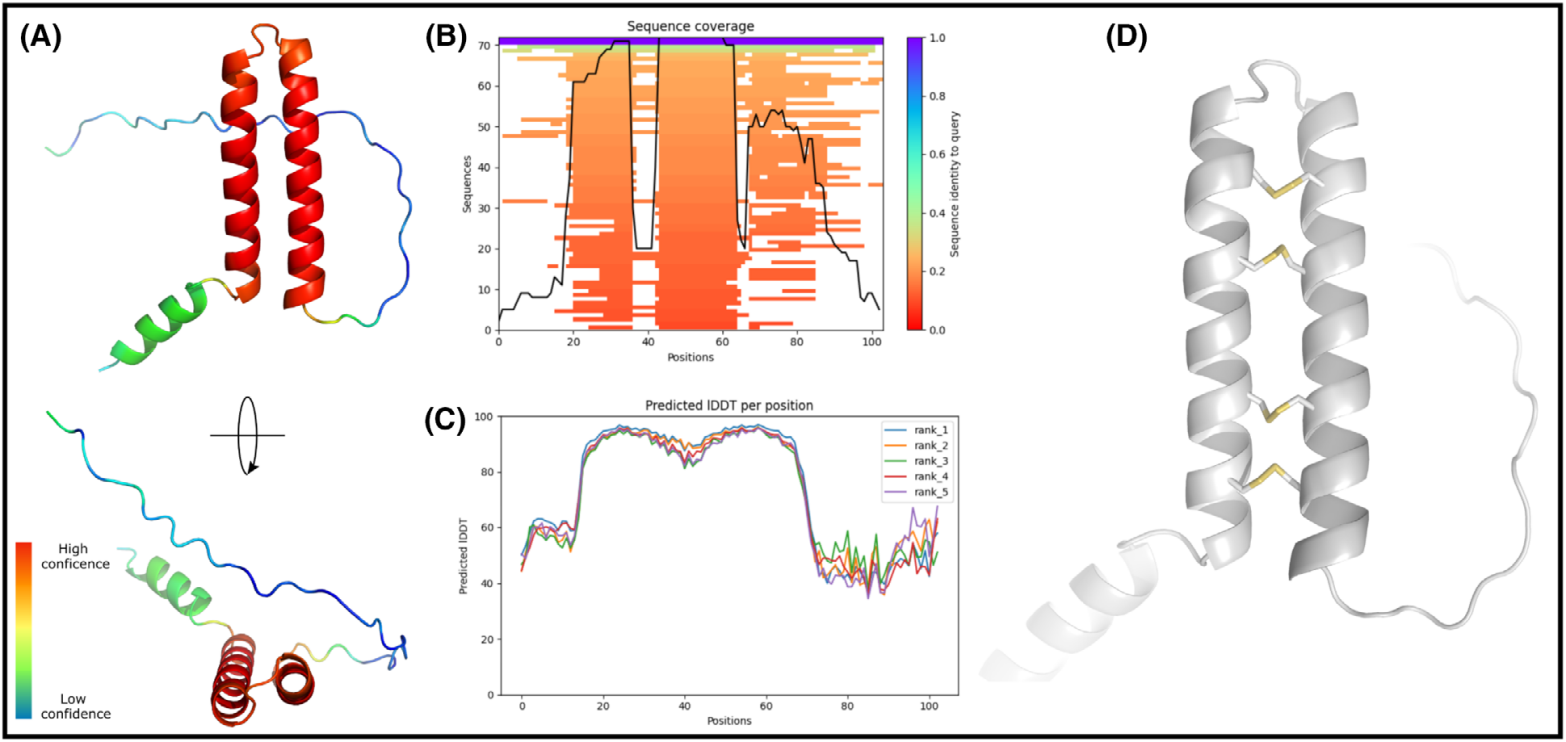
Prediction of the accripin11 3D structure with AlphaFold2. (A) Two orientations of the best‐predicted accripin11 model. Residues have been colored according to their score of confidence. (B) Number of homologous sequences identified per position – 30 to 100 sequences are required for best performance prediction. (C) Score of confidence (IDDT) for the best five predicted models. IDDT for each residue are displayed, and higher values correspond to better prediction score. (D) Close‐up view of the antiparallel helix bundle showing the formation of four disulfide bridges predicted by AlphaFold2.

The analysis of accripin11's putative three‐dimensional structure via AlphaFold2 (Fig. 4A) indicates that the protein is comprised of three consecutive alpha helices and a disordered C‐terminus (~ 35 residues). Further analysis of the sequence alignments used for model predictions shows that the N terminus (first 20 residues) and C‐terminus (last 35 residues) have highly unusual amino‐acid composition, which prevent reliable identification of homologous targets (Fig. 4B) and therefore a low confidence in the prediction for these two regions (Fig. 4C). Only helices 2 and 3 (in red Fig. 4A) show a score of confidence high enough to make interpretations regarding the predicted fold. Interestingly, these two helices are organized in an antiparallel manner and held together by four disulfide bridges that apparently stabilize the helix bundle (Fig. 4D).

Alphafold2 was also used for predicting the 3D structures of the six putative molluscan shell proteins that exhibit homology with accripin11 including the same cysteine pattern. As shown in Fig. S3, the best‐predicted models (ranked 1) demonstrate that all six sequences possess the same structural motif consisting in the antiparallel alpha‐helix containing 4 disulfide bonds. All six C‐termini are disordered.

### Overexpression of recombinant accripin11

**Fig. 5:**
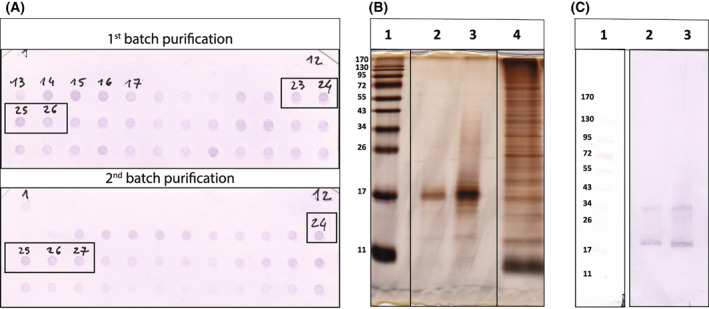
Purification of overexpressed accripin11 by preparative gel electrophoresis. (A) Dot blot of the first 48 fractions eluted from two batches of preparative SDS/PAGE. The dots were stained with the anti‐strepTag2 antibody. Tubes 23–26 from 1st batch and tubes 24–27 from 2nd batch (boxed in black) were pooled, respectively. (B) Silver‐stained SDS/PAGE gel (15% acrylamide) of pooled tubes of 1st batch (lane 2) and of 2nd batch (lane 3). Lane 1 is molecular weight standards and lane 4, partially purified accripin11. (C) Western blot (12% acrylamide) of pooled fractions of the two batches stained with anti‐strepTag2 antibody (lane 2 and 3 for 1st and 2nd batch, respectively). Lane 1, molecular weight standards, same as for B.

Accripin11 was overexpressed and purified by ProteoGenix via affinity chromatography (using a StepTag2 tag and StrepTactin resin) under native conditions. When tested on silver‐stained gel (Fig. 5B, lane 4), we found this initial purification to be incomplete (Fig. 5B, lane 4). Consequently, the extract was purified again by preparative gel electrophoresis in two batches and pure Accripin11 was detected on dot‐blot (Fig. 5A) with anti‐StrepTag2 antibody, in tube 23–26 (1st batch) and 24–27 (2nd batch). We verified that this preparative purification yielded accripin11 by performing proteomics on the resulting extracts: this fully confirmed the presence of accripin11.

The purity of the pooled fractions of both batches was tested on silver‐stained gel (Fig. 5B, lanes 2 and 3) and western blot (Fig. 5C, lanes 2 and 3): in the first case, one unique band around 17 kDa was visualized on the gel, while the western blot shows predominantly one band at the same height, but also another thin band around 34 kDa, which may represent a dimer of accripin11. It is noteworthy that this protein exhibits anomalous gel migration as its predicted size is 11 kDa.

### Layer‐specificity of accripin11

We investigated whether accripin11 was spatially restricted to the calcitic prisms of the shell of *P. nobilis*, or if it could also be found in the nacreous layer. To this end, we extracted the ASM and AIM of the nacreous layer, as for the prisms layer. All six fractions, including the four prisms extracts (ASM_p_1, ASM_p_2, AIM_p_1, AIM_p_2) and the two nacre ones (ASM_n_, AIM_n_), were tested by proteomics for the presence of accripin11. The results are summarized in Table 1. In addition, the list of peptides identified in each of the six extracts is shown in Table S1. In brief, all 6 extracts contain accripin11 but in varying abundance: while ASM_p_1 and ASM_p_2 contain respectively 40 and 36 peptides representing 93% of the sequence of mature protein (without its signal peptide), ASM_n_ contains a single short peptide covering only 6% of the sequence. AIM_p_1 and AIM_p_2 contain also accripin11 with a relatively good coverage (58, 63%), but with a limited number of peptides (10 and 7, respectively); at last, AIM_n_ contains only three accripin peptides, covering 40% of the sequence. Although not strictly quantitative, these data suggest that accripin11 is concentrated in the two soluble prism matrices, less concentrated in the insoluble prism matrices, and very poorly concentrated in the two nacre matrices.

**Table 1:** Presence of accripin11 in the different shell layer extracts via proteomics. The experiment was performed in soluble and insoluble extracts of prismatic layer (ASM_p_1, AIM_p_1), isolated prisms (ASM_p_2, AIM_p_2) and nacreous layer (ASM_n_, AIM_n_). The number of peptides provided by this protein in each extract along with the sequence coverage by these peptides is also included.

Shell layer	Cleaned prismatic layer		NaOCl‐isolated prisms		Cleaned nacreous layer	
Extract	ASM_p_1	AIM_p_1	ASM_p_2	AIM_p_2	ASM_n_	AIM_n_
Detected Y/N	Y	Y	Y	Y	Y	Y
Number of peptides	40	10	36	7	1	3
Sequence coverage (%)	93	58	93	63	6	40

**Fig. 6:**
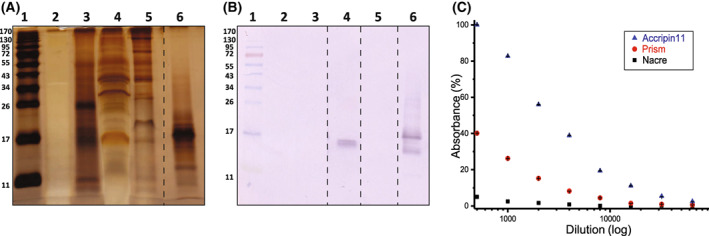
Presence of accripin11 in different shell extracts. (A) SDS/PAGE. (B) Western blot. A and B correspond to 15% acrylamide gels. In A and B, the samples occupy the same lanes; lane 1: molecular weight standards; lane 2: ASM_n_; lane 3: AIM_n_; lane 4: ASM_p_1; lane 5: AIM_p_1; lane 6: purified accripin11. The western blot was incubated with anti‐accripin11. Note that the recombinant purified accripin11 tends to degrade in a component of lower molecular weight, visualized by the antibody. The dashed lines between lane 5 and 6 in A and between lanes 3, 4, 5 and 6 in B indicate that the lanes were spliced together. (C) ELISA test of ASM_n_, ASM_p_1 and accripin11 with serial dilutions of anti‐accripin11 antibody serum ranging from 500× to 64 000×. Absorbance values at 405 nm were normalized to highest value (accripin11, 500× dilution antibody) corresponding to 100% reactivity.

We also performed a western blot (Fig. 6B) under the same conditions as for the gel (Fig. 6A). The blot, treated with anti‐accripin11 antibody, revealed one band in the ASM_p_ at approximately 17 kDa (Fig. 6B, lane 4) corresponding to a blurred negatively stained band in the silver‐stained gel (Fig. 6A, lane 4). No band was identified in LS‐AIM_p_ (Fig. 6B, lane 5) neither in ASM_n_ (Fig. 6B, lane 2) nor LS‐AIM_n_ (Fig. 6B, lane 3). This suggests that accripin11 is particularly concentrated in the acetic acid soluble matrix of the prisms but poorly concentrated or absent from the other extracts. We cannot exclude that, as polymerized or crossed‐linked form, this protein may be present in the insoluble matrices of both the prisms and the nacre, since it was identified by proteomics in these two fractions.

As the solubility of accripin11 limited our options, ELISA tests were performed only on the two acetic acid‐soluble matrices, with recombinant accripin11 acting as a positive control (Fig. 6C). The graph shows that the anti‐accripin11 antibody cross‐reacted with ASM_p_ but not with ASM_n_ tested under the same conditions and at the same concentration (200 ng per well), suggesting that accripin11 is concentrated in the prism matrix, but is absent (or very rare) in the nacre matrix. Quantification by ELISA of accripin11 in the prism soluble matrix was performed with a calibration curve with the target protein tested at known serial concentrations (Fig. S4). The ELISA indicates that accripin11 may represent around 12% of the prism ASM, making it a major soluble protein of the prismatic matrix.

### *In vitro* crystallization in the presence of recombinant accripin11

**Fig. 7:**
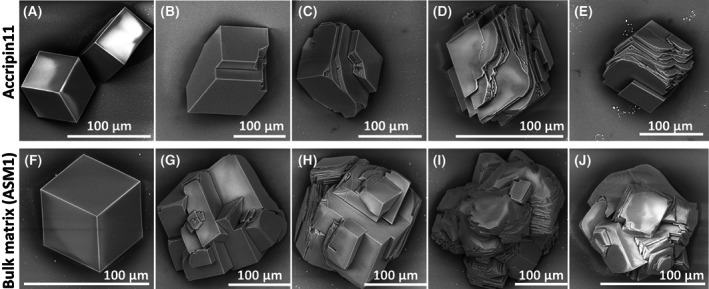
*In vitro* CaCO_3_ crystallization assay. (B–E) Test in the presence of accripin11. (G–J) Test in the presence of the bulk prism soluble matrix, ASM_p_1. Both were tested in the same conditions and order of concentrations at 0.5, 1, 2 and 4 μg·mL^−1^. A and F represent blank test (in the absence of accripin11 and ASM_p_1). Scale bar: 100 μm for all images.

The interaction of purified accripin11 and bulk shell matrix (ASM1) with the precipitation of calcium carbonate crystals grown *in vitro* was investigated by scanning electron microscopy (SEM) (Fig. 7). When no protein is added, that is, blank, in both cases, the typical rhombohedral calcite crystals with smooth crystal faces were obtained (Fig. 7A,F).

When accripin11 was tested, we observed a gradual change in morphology, which is concentration‐dependent (Fig. 7B–E). However, this change was gradual: even at high concentration (Fig. 7E), the produced crystals still exhibited sharp edges and few rounded angles. In contrast, when ASM1 (Fig. 7G–J) was tested for comparison, the alteration of crystal morphology was already observed at 0.5 μg·mL^−1^, that is, polycrystalline aggregates were observed with rounded edges (Fig. 7G). This alteration was accentuated at 1 μg·mL^−1^ (Fig. 7H). Starting from 2 μg·mL^−1^ (Fig. 7I), the effects were drastic: we observed fully rounded polycrystalline aggregates. With respect to crystal size, we noticed differences between the two extracts: accripin11 did not induce any size modifications in the tested concentration range, while we observed a size increase up to 2 μg·mL^−1^ (Fig. 7I) and then a size decrease in the case of ASM1 (Fig. 7J). We interpret the size decrease as an inhibitory effect of ASM1.

## Discussion

In this paper, we describe a novel protein derived from the shell matrix of the Mediterranean fan mussel, *P. nobilis* that we have named accripin11. It is a small, secreted protein of 103 amino acid residues, with a theoretical isoelectric point of 6.7. This makes accripin11 not particularly acidic, in contrast to a large cohort of proteins extracted from mollusk shells, and more generally, from metazoan calcium carbonate skeletons [7, 26]. However, the protein contains an exceptionally acidic C‐terminus with 10 acidic residues out of 17. Although accripin11 might be ubiquitous in the shell, both proteomics and the use of an anti‐accripin11 antibody show that this protein is most abundant in the prismatic layer, and particularly in the acetic acid‐soluble fraction, a fact that is in agreement with the overall hydrophilicity of accripin11. Western blot failed to detect accripin11 in the Laemmli‐soluble acetic acid‐insoluble fraction of both prismatic and nacreous layers, but proteomics identified accripin11 in the acetic acid‐insoluble fraction of the prisms. This latter comprises the Laemmli‐soluble acetic acid‐insoluble fraction and the most insoluble fraction, which was not analyzed further. The apparent discrepancy between western blot and proteomics may be explained as follows: beside being associated with the acetic acid‐soluble fraction, accripin11 is also present in the most insoluble matrix, the fraction that cannot be solubilized further by Laemmli sample buffer, that is, that cannot be analyzed further via techniques such as western blot or ELISA. If so, this means that accripin11 may polymerize into large insoluble units, or that it extensively crosslinks with other components to form a completely insoluble matrix.

The sequence of accripin11 is characterized by a short basic N terminus, a cysteine‐rich domain, a hydrophobic alanine‐rich domain and a very acidic C‐terminus. This primary structure is rather unusual: while 80% of the sequence is ordered and consists of three successive alpha‐helices in the cysteine‐rich domain, the terminal “tail” is disordered and highly acidic. The presence of such a terminal domain may explain why accripin11 exhibits an anomalous migration in SDS/PAGE gels: both the recombinant protein (which contains the Strep2 tag) and the protein from the soluble prism matrix show delayed migration (often referred to as “gel shifting”) under denaturing conditions (from 11 to 17 kDa). According to Tiwari and coworkers, proteins with acidic domains always exhibit an overestimated apparent molecular weight. These authors explain this phenomenon by the fact that highly acidic domains electrostatically repel SDS, resulting in insufficient SDS binding and consequently lowered electrophoretic mobility [27]. We cannot exclude that incomplete unfolding of accripin11 may be another cause for anomalous migration in a denaturing gel, but because of the concentration of mercaptoethanol used, this explanation is less likely.

Because accripin11 only exhibits sequence similarity with putative shell proteins that have not been functionally tested, its functions(s) in biomineralization remain unknown. Consequently, it belongs to the growing collection of shell proteins of “unknown function,” similar to MRNP34 [17], or to upsalin [23]. However, when tested *in vitro*, we observed that recombinant accripin11 exerts a relatively strong effect on the precipitation of calcium carbonate, in spite of its almost neutral isoelectric point. Whether this effect is simply an artifact due to the sequence peculiarities of accripin11, or whether it is an accurate representation of its *in vivo* function remains unknown, and a proper answer will only be given via gene knock‐down/CRIPR‐Cas9 technologies. Already perceptible at a low protein concentration (0.5 μg·mL^−1^), the interference effect increases proportional to the protein concentration. We noticed however that the interaction of accripin11 alone with growing crystals is less effective than the effect of the bulk soluble matrix. This latter represents a pool of numerous proteins, including highly acidic ones such as caspartin, which was found to be effective in the interference as well as inhibition tests [28]. This suggests that synergistic or additive effects occur during the *in vitro* growth of calcium carbonate crystals when a bulk soluble matrix is used instead of a purified protein.

In a schematized view (Fig. 4A), the Asp‐rich C‐terminus of accripin11 may bind the positively charged surface of growing calcium carbonate nuclei via electrostatic interactions, while the more hydrophobic domain may be repelled and protrude from the surface, interfering with the normal growth of crystallites by disrupting the movements of lattice ions to the crystals, as has been suggested for synthetic poly‐aspartic acid peptides containing a polyalanine domain [29]. In past simulations, it was found that a short polyalanine (i.e., hydrophobic) domain of 8 or so residues would be sufficiently large at about 3 nm to interfere with the zone of attraction between lattice ions and surface charges [29, 30]. In this view, the hydrophobic domain would then control the access of lattice ions to crystal surfaces. Accripin11 exhibits such short hydrophobic motifs in the first half of its sequence, for example, between residue 12 (2nd methionine) and 18 (leucine) and between the second cysteine (position 25) and phenylalanine (position 39). However, we cannot exclude other functions of the N terminus and/or the central domain; these may bind other macromolecular partners of the matrix via the three alpha helices. If this is the case, accripin11 may then act as a true “linker” between the mineral surface and the organic framework.

In our representation, the unstructured C‐terminus interacts mainly with calcium carbonate minerals (crystallized or amorphous) but we can also envisage its folding to be induced by a partner macromolecule. It is interesting to note that accripin11 is not the only example of a relatively short shell‐associated protein with an acidic tail; as revealed by our blast searches, this property is shared with all other putative members of the family and with the two members of the prismin “family,” (prismin1 and 2 [31]). These latter are two polypeptides of about 4 kDa identified in the prismatic layer matrix of the Japanese pearl oyster *Pinctada fucata*. Like accripin11, prismin 1 and 2 exhibit apparent molecular weights on Tris‐tricine gels higher than their respective sequences suggest. These two polypeptides belong to a protein family that is apparently unrelated to accripin11. However, their discoverers suggest that their acidic C‐termini have the ability to interact with the surface of calcium carbonate crystals.

**Fig. 8:**
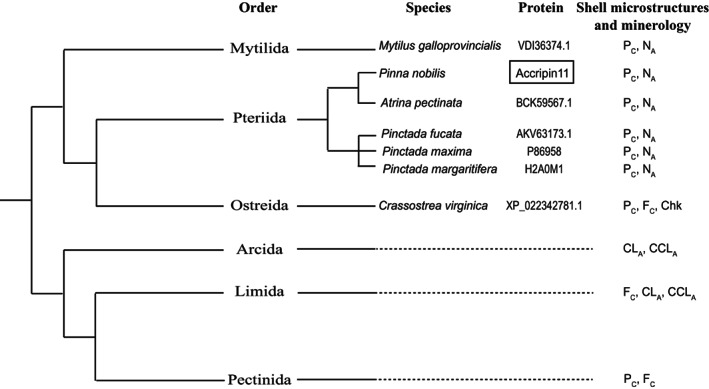
Simplified phylogenetic tree of the Pteriomorphia subclass, adapted from [35]. Except accripin11, the members of the protein family are indicated by their UniProt accession numbers. The right column indicates the shell microstructures and mineralogy identified in the six orders. P, prismatic; N, nacreous; F, foliated; CL, crossed‐lamellar; CCL, complex crossed lamellar; Chk, chalky. In subscript characters, C, calcite; A, aragonite.

The sequence similarity of accripin11 along with six other putatively related proteins suggests a common ancestry of these proteins that appears to be restricted to the Pteriomorphia. All of these sequences are defined by their patterns of cysteine, proline and arginine residues and the overall high‐sequence similarities, the distribution of hydrophobic and hydrophilic motifs along their sequence and their acidic C‐termini. Furthermore, Alphafold2 predictions indicate that all seven contain the antiparallel alpha helix, a conserved structure that most likely has an important function in biomineralization. In spite of the significant morphological diversity of the Pteriomorphia, this major bivalve subclass, considered to be monophyletic [32, 33] is comprised of six orders including the Arcida, Pectinida, Limida, Ostreida, Mytilida and Pteriida [34]. The seven proteins of the accripin family belong without exception to representatives of the three last orders, namely Ostreida (*C. virginica*), Mytilida (*M. galloprovincialis*) and Pteriida (with the three *Pinctada* species, belonging to Pterioidea superfamily, and *A. pectinata* + *P. nobilis*, belonging to the sister superfamily, Pinnoidea). Interestingly, a relatively recent large‐scale phylogeny of bivalves [35] groups these three orders together (Fig. 8), making them the sister group of the Arcida‐Limida‐Pectinida clade. In this phylogeny, Ostreida and Pteriida are sister groups and, together, are the sister group of Mytilida.

Although there is no consensus on the branching pattern of the six pteriomorphian orders, both palaeontological [36] and more recent molecular clock data [37] indicate that these orders diverged in the Paleozoic era, between the Silurian (> 430 million years ago) and the Devonian (> 385 million years ago). Therefore, the most likely scenario is that the seven accripin11 orthologs are derived from a single “Lower Paleozoic” ancestor that existed before the Mytilida/Ostreida/Pteriida split. Under this scenario, accripin constitutes a clear example of a lineage‐restricted shell‐forming protein. In the future, as more bivalve genomes become available, it will be fascinating to check whether members of this family are also present in the three other pteriomorphian orders, namely Arcida, Limida and Pectinida (blast searches against these lineages currently reveal no hits against accripin11). In contrast to *Mytilus*, *Crassostrea*, *Atrina*, *Pinna* and *Pinctada*, representatives of these three orders do not possess an outer shell layer made of calcitic prisms, but rather, either an outer layer that is foliated calcite (Limida, Pectinida) or crossed‐lamellar aragonite (Arcida) [38]. This intriguing observation suggests that accripin‐related proteins may be functionally involved in the deposition of prismatic shell microstructures.

## Methods

### Earlier work for identifying accripin11

In 1996, mantle tissue was collected (0.5 g) from actively calcifying, juvenile *P. nobilis*, grown in aquaria (B. De Gaulejac) as previously described [39]. The amplified cDNA expression library (LambdaZap) made from this tissue library was antibody‐screened. Screens with antibodies elicited against the soluble nacre matrix of *P. nobilis* led to the identification of mucoperlin [39, 40]. A similar operation performed some years later with antibodies elicited against the soluble prism matrix generated 15 positive clones, which were isolated, rescreened to purity and sequenced (Eurofins Genomics, Ebersberg, Germany). The sequence of one of these inserts contained a 363 bp ORF, a 441 bp 3′ UTR and a poly(A) tail. This ORF encoded an undescribed putative shell protein of 121 amino acids, which we initially named CSP3 [26] and have here renamed accripin11 and fully described.

### Sample collection: fresh tissues and shell materials

A second series of mantle tissue collections was performed in the spring of 2017, requiring the authorization of the DDTM (Direction Départementale du Territoire et de la Mer of Alpes Maritimes department, Arrêté Préfectoral n° 2017‐459). Ten juvenile *P. nobilis* individuals (from 15 to 30 months) were collected between 5 and 8 m by SCUBA diving, at the Baie de Villefranche‐sur‐Mer in May 2017. Shells were carefully taken together with their byssus and the substrate attached to them. All sampling and operations were performed according to the appropriate ethics rules and regulations. Sampling mission information and metadata are stored in dat@UBFC portal of the Observatoire des Sciences de l'Univers (OSU) Theta, Besançon, France at: https://search‐data.ubfc.fr/search.php?s=Pinna+nobilis.

Individual mussels were placed in a seawater‐filled tank on the boat, then transferred and acclimated in a large basin at the biological station, and fed twice a day with Seachem Reef Phytoplankton™ containing a mixture of algae (*Thalassiosira weissflogii*, *Isochrysis* sp., *Nannochloropsis* sp.), protein hydrolysate with carotenoids, citric acid, carboxylic acid, methyl paraben, sodium propionate. After 5 days, some animals were sacrificed. Valves were carefully opened, by cutting the adductor muscle with a scalpel. Only the outer border of the mantle characterized by several folds (which is supposed to contribute only to the secretion of the prismatic layer, including the spines) was sampled in addition to byssal glands, foot and gills. All tissues were immediately frozen in liquid nitrogen. Unused living specimens were transferred back to the Villefranche‐sur‐Mer bay and returned to their original biotope.

The shells of sacrificed animals were carefully cleaned to remove epibionts and kept for further matrix extraction.

### Transcriptomics

Total RNA was extracted from the mantle of two individuals using QIAzol (#79306; Qiagen GmbH, Hilden, Germany) following the manufacturer's instructions. The RNA extractions were quantified with a Nanodrop and qualified by agarose gel electrophoresis before being sent to the NGS‐Service for Integrative Genomics (Göttingen) for library preparation and sequencing. Paired‐end libraries were constructed and sequenced for 250 bases from both ends on the Illumina HiSeq2500 platform. Raw Illumina reads were processed and assembled as previously described [41]. We particularly focused on the 1417 bp‐long transcript R20673250 that contains a 363 bp‐long ORF encoding accripin11.

### Prism matrices extraction and mono‐dimensional gel check

A cleaned shell valve of a 28‐ to 30 –month‐old specimen was used for the extraction of the prism matrix. In brief, the upper two‐third of the shell (which exclusively contains the prismatic layer) was cut into fragments with a diamond saw. The fragments were sonicated in diluted NaOCl (1% active chlorine) for 5 min, rinsed with water, 70% alcohol, air‐dried and roughly crushed. The cleaned fragments were divided into two batches: one for extracting the whole organic matrix and the other for extracting only the intra‐prismatic organic matrix. The first batch was powdered by using a mortar and pestle grinder to a particle size below 200 μm (sieving). The second batch was bleached in NaOCl for 2 days under constant rotation (Speed 25 r.p.m.) to isolate prisms by dissolving the peri‐prismatic organic sheaths. Prisms were then collected by centrifugation, the NaOCl discarded, and the suspension was rinsed several times with Milli‐Q water and dried [28].

Both powdered (Batch 1 = 15.8 g) and bleached (Batch 2 = 12.99 g) samples were decalcified overnight, by titrating with cold diluted acetic acid (10% v/v) to obtain the full prismatic and intra‐prismatic organic matrices, respectively. The obtained clear solution from both batches was then centrifuged (3900 ***g***, 30 min) to separate an Acid Soluble Matrix of the prisms (ASM_p_) from an Acid Insoluble Matrix of the prisms (AIM_p_). The AIMs of the two batches (AIM_p_1 and AIM_p_2) were rinsed with Milli‐Q water and freeze‐dried. ASM_p_1 and 2 were further ultrafiltered by using 3 kDa cut‐off membrane (volume reduction to approx. 10 mL), and the concentrated solutions were desalted by dialyzing (Spectra/Por6 dialysis pre‐wetted RC tubing, molecular weight cutoff 1 kDa) in 1 L Milli‐Q water, with at least five water changes. These salt‐free solutions were then freeze‐dried. Aliquots were denatured with Laemmli sample buffer. The AIMs did not fully dissolve but the solubilized fractions were named LS‐AIM (Laemmli‐Soluble Acid Insoluble Matrix). ASMs and LS‐AIMs were run on hand‐casted 15% acrylamide mono‐dimensional mini‐gels (Bio‐Rad Laboratories, Hercules, CA, USA) according to the manufacturer's instructions. The gels were stained with silver nitrate [42].

### Proteomics and subsequent *in silico* analysis

MS/MS analyses were conducted on the four unfractionated bulk matrices, ASM_p_ and AIM_p_, after a short migration in an acrylamide gel according to a published procedure [20]. Database searches were carried out using mascot version 2.4 and 2.5 (MatrixScience, London, UK) using the *P. nobilis* transcriptome.

The accripin11 sequence, both deduced from the analysis of the 1417 bp‐long transcript R20673250 and from proteomics, was analyzed for its physico‐chemical parameters, including isoelectric point, molecular weight, amino acid composition, by using the Protparam [43]. Its signal‐peptide was identified by signalp‐6.0 [44]. We performed standard blastp searches against GenBank (https://blast.ncbi.nlm.nih.gov) and we used the Pattinprot search tool from PRABI (Pôle Rhône‐Alpes de Bioinformatique, Lyon, France) to identify cysteine patterns similar to that of accripin11.

### AlphaFold2 simulations

3D model predictions of accripin11 were obtained using first, a local installation of AlphaFold2 [45] and later, the online version of ColabFold [46] from which alignment statistics and prediction scores were extracted. In addition, 3D model predictions were performed with the six putative molluscan shell proteins that contain the same cysteine pattern and that were found to exhibit sequence similarity with accripin11. Figures of the best‐predicted model were prepared with the pymol Molecular Graphics System version 2.4.0 (Schrödinger platform, Mannheim, Germany).

### Overexpression of recombinant accripin11 and purification

Overexpression work was performed by the company ProteoGenix (Schiltigheim, France). In brief, the cDNA coding for mature accripin11 (without signal peptide) was chemically synthesized with optimization for *Escherichia coli* expression. It was subsequently cloned into a pT7 expression vector, containing a StrepTag2 tag in its C‐terminus, according to the manufacturer's instructions. The subcloned DNA insert encodes a recombinant protein that is slightly longer than the natural one (Fig. S5) as it includes an MG dipeptide at the N terminus (from the pT7 vector) and the 10 AA‐long StrepTag2 tag (SAWSHPQFEK) at the C‐terminus. The culture growth conditions were as follows: growth at 37 °C until OD600 nm > 0.5, followed by induction of overexpression with IPTG (1 mm final concentration, induction time from 1 to 4 h). Cells were harvested by centrifugation, disrupted by sonication in native buffer and tested on a standard SDS/PAGE gel (not shown). Relatively low expression was observed. The protein was purified from the supernatant by affinity chromatography as follows: the supernatant was allowed to bind to a StrepTactin resin, which was subsequently washed with TBS. The bound extract was then eluted with desthiobiotin. As the purification was far from optimal as revealed by silver‐stained gels, we decided to purify it further by preparative electrophoresis (Bio‐Rad; model 491 Prep Cell) on a 12% acrylamide gel, according to a protocol developed by one of us [39, 47]. Two successive purifications were performed from two equal batches. The protein was detected by dot‐blot on the 80 different eluted fractions owing to an anti‐StrepTag2 antibody, diluted 1/1000 (StrepMAB‐Classic, ref. 2‐1507‐001; IBA Lifesciences, Göttingen, Germany). The tubes containing the eluted recombinant protein (Tubes 23–26 for batch 1, tubes 24–27 for batch 2) were pooled and the pooled fractions, dialyzed against milli‐Q water (Spectra/Por6 dialysis pre‐wetted RC tubing, molecular weight cutoff 1 kDa), before being freeze‐dried. The extract was quantified by weighing the lyophilizate: around 300 μg of pure protein was obtained. An aliquot (about 10 μg) was sent to 3P5 platform for proteomic analysis, to ensure the “accripin11 nature” of the extract.

The freeze‐dried pellet was dissolved again in Milli‐Q water, and an aliquot was tested on a SDS/PAGE gel as described above (with silver staining), and on western blot. In this last case, we employed a procedure currently used in our lab [28] using Mini‐Trans Blot module (Bio‐Rad) and a 90 min. Transfer at 110 mA (constant). After blocking, the membrane was incubated with anti‐Strep2 antibodies, before being revealed either with the chemiluminescent substrate CDP‐Star (Sigma‐Aldrich Chimie SARL, Saint‐Quentin Fallavier, France; ref. C0712‐100ML) or with SIGMA*Fast* BCIP®/NBT tablets (Sigma‐Aldrich; ref. B5655‐25TAB).

### Polyclonal antibodies against synthetic peptides of accripin11

Since the quantity of pure recombinant accripin11 was too low to generate antibodies and to develop in parallel *in vitro* assays, we chose to produce a polyclonal antibody from two synthetic immunogenic peptides corresponding to the central (KDCAQQCTRDRETCFG, residues 65–80) and to the C‐terminal part (TAAPKPAKEPSSADD, residues 97–111) of the accripin11 sequence. The whole procedure (peptide synthesis + antibody production) was performed by Eurogentec (Seraing, Belgium). The synthesized peptides were coupled to a carrier (KLH for the 1st central peptide, OVA for the 2nd C‐terminal one) and injected in two white rabbits (SY3579, SY3580), according to a speedy 28‐day immunization protocol (contract FR10162), including injections at 0, 7, 10 and 18 days, and bleedings at 0 (pre‐immune serum), 21 (medium) and 28 (final) days. The titers of second and third bleed antiserum were determined by ELISA test with pre‐immune serum used as negative control, as previously described [28, 39].

### Localization of accripin11 in the nacreous layer

To investigate whether accripin11 was either exclusively associated with the prismatic layer of *P. nobilis* shell or also present in the nacreous layer, we employed two strategies: (a) searching for accripin11 in nacre extracts via qualitative proteomics and (b) testing the presence of accripin11 in both extracts with the polyclonal anti‐accripin11 antibody. In both cases, about 8‐g portions of the nacreous layer of a cleaned shell (of a juvenile specimen) were mechanically isolated from the prismatic layer, powdered and decalcified overnight at 4 °C with cold acetic acid (10% v/v), similarly to the prismatic layer (see above). These extractions generated an acetic acid‐insoluble (AIM_n_, 67.4 mg, i.e., 0.85% of the powder weight) and acetic acid‐soluble (ASM_n_, 4.6 mg, i.e., 0.058% of the powder weight) nacre fractions. In strategy 1, aliquots of these two extracts were analyzed via proteomics (3P5 platform) identically to the prism extracts, and the accripin11 sequence was searched via mascot. According to strategy 2, the soluble fractions of both prisms (ASM_p_) and nacre (ASM_n_) were tested by ELISA according to two approaches, and by western blot. For the first ELISA approach the antigens concentrations were kept constant (200 ng per well) and the anti‐accripin11 antibody was 2‐fold serially diluted from 1/500 to 1/64 000 (eight dilutions). Purified accripin11 was used as a positive control at 100 ng per well. Each point was tested in triplicate. For the second ELISA approach, the two soluble fractions (ASM_n_, ASM_p_) were 2‐fold serially diluted, from 800 to 6.25 ng per well and the antibody dilution was kept constant (1/1000). In parallel, on the same plate, a calibration curve was generated with accripin11 using concentrations varying from 100 to 0.78 ng per well. Each point was tested in triplicate. For the western blot, Laemmli‐denatured preparations of ASM_p_, AIM_p_, ASM_n_, AIM_n_ and purified accripin11 were run on a 15% acrylamide gel and electro‐transferred as indicated above. After blocking, the membrane was incubated overnight with anti‐accripin11 antibody, diluted 1/1000. After rinsing (TBS/Tween 20), the membrane was incubated 90 min with GAR‐AP conjugate diluted 1/30 000 (Sigma‐Aldrich; A3687‐1ML), extensively rinsed and the color developed either by chemo‐luminescent staining (CDP‐Star) or by SIGMA*Fast* BCIP®/NBT tablets.

### *In vitro* crystallization

Directly following its purification, the interaction of recombinant accripin11 with the growth of calcite *in vitro* was tested in the calcium carbonate crystallization assay (diffusion method), according to the initial Albeck et al. protocol [48] with some modifications (as described in ref. [49]). We did not remove the StrepTag2 tag from the recombinant accripin11 as it is regarded to be biologically inert, proteolytically stable and unlikely to interfere with the folding or bioactivity of recombinant protein [50]. The effect of recombinant accripin11 on crystallization was compared to that of the complete soluble prism matrix (ASM_p_1). Increasing concentrations of accripin11 (top row) and of ASM_p_1 (bottom row), ranging from 0.5 to 4 μg·mL^−1^ in 10 mm filtered sterile CaCl_2_, were applied to 16‐well culture slides (Lab‐Tek, Nunc/Thermo Scientific, Rochester, NY, USA; 200 μL per well). The plate with a pierced top was kept in a desiccator under vacuum in the presence of ammonium bicarbonate (NH_4_HCO_3_) crystals and maintained at 4 °C. Blanks were tested with CaCl_2_ solution alone. After 72 h, the solutions were carefully removed from the wells (with a blunt end needle connected to a Millipore vacuum pump), and the plate was dried. The glass plate of the slide was dissociated from the well spare part and directly observed under a Hitachi TM1000 Tabletop Microscope without carbon coating. This experiment was repeated three times to ensure homogeneity of the results.

## Conflict of interest

The authors declare no conflict of interest.

## Author contributions

BK was involved in investigations, methodology, data analysis, original draft and conception of figures; DJJ was involved in transcriptomics, data analysis, review, validation and editing; SE and PBC were involved in 3D modeling and writing; SM was involved in sample collection; CB was involved in proteomics analysis and writing; JT was involved in conception of macro‐photographs and funding acquisition for proteomics (Recolnat); FI was involved in protein overexpression and funding acquisition for recombinant protein overexpression (IABECA); MJH was involved in funding acquisition for transcriptomics (DFG) and writing; DV was involved in conceptualization and writing; JP was involved in conceptualization, funding acquisition (ANR) and writing; FM was involved in conceptualization, funding (SRO, EMBRC), project administration, investigations, methodology, data analysis, original draft, supervision, validation, review and editing.

## Supporting information


**Fig. S1.** Nucleotide sequence of the contig (>Pnobilis_R20673250) encoding accripin11. The amino acid sequence of accripin11 is shown under the nucleotide sequence in one‐letter symbols (in blue): the first 18 amino acids in red represent the signal peptide. This amino acid sequence was found in the longest open reading frame of the translation 2F.
**Fig. S2.** Alignment of the cysteine pattern (shaded blocks) with that of one zinc finger protein (Swiss‐Prot accession number Q8R151) and one keratin‐associated protein (Swiss‐Prot accession number Q64507) of *Mus musculus*.
**Fig. S3.** 3D structure prediction with AlphaFold2 of the 6 putative molluscan shell sequences that were found to be homologous to accripin11: (A) *Atrina pectinata*, (B) *Crassostrea virginica*, (C) *Mytilus galloprovincialis*, (D) *Pinctada fucata*, (E) *Pinctada maxima* and (F) *Pinctada margaritifera*. Each of these predictions shows the two antiparallel alpha helices. With the exception of the protein C, all of them exhibit a disordered C‐terminus.
**Fig. S4.** Quantification of Accripin11 by ELISA in ASM_p_1 and ASM_n_ extracts. The curve was obtained with recombinant Accripin11, serially diluted (100 to 0.7 ng) and tested with the anti‐accripin11 antibody, diluted 1000 times. (A) Calibration curve; the red line represents the linear fit and the insert table includes the linear equation and its parameters. (B) List of absorbance values obtained with ASM_p_1 and ASM_n_, both tested at concentrations ranging from 800 to 6.25 ng per well. The absorbance value of ASM_p_1 at 200 ng/well is almost equal to the value of accripin11 at 25 ng/well. Note that all absorbance values measured in ASM_n_ correspond to blank values, indicating the absence of accripin11 in the extract.
**Fig. S5.** Complete amino acid (AA) sequence of recombinant accripin11 with the StrepTag2 tag. The first two AAs (in red) belong to the pT7 expression vector. AAs in blue (from position 106 to position 115) represent the StrepTag2 tag.

**Table S1.** List of peptides identified by MASCOT for accripin11 in the four matrices of prismatic layer (ASM_p_1, AIM_p_1, ASM_p_2 and AIM_p_2) and in the two nacre matrices (ASM_n_, AIM_n_).

## Data Availability

The transcriptomic data used in this manuscript have been submitted to the SRA database under the BioProject accession number PRJNA887567 at NCBI (https://www.ncbi.nlm.nih.gov/sra/PRJNA887567). The sequence of accripin11 can be found in GenBank under the accession number OP610121.
