# Supplementary material for: Molecular characterization of accripin11, a soluble shell protein with an acidic C‐terminus, identified in the prismatic layer of the Mediterranean fan mussel Pinna nobilis (Bivalvia, Pteriomorphia)
Source: FEBS Open Bio. 2022 Dec 3;13(1):10–25. doi: 10.1002/2211-5463.13497 (PMC9808598; doi:10.1002/2211-5463.13497)
Supplement: Supplementary file 1 — Fig. S1. Nucleotide sequence of the contig (>Pnobilis_R20673250) encoding accripin11. The amino acid sequence of accripin11 is shown under the nucleotide sequence in one‐letter symbols (in blue): the first 18 amino acids in red represent the signal peptide. This amino acid sequence was found in the longest open reading frame of the translation 2F. Fig. S2. Alignment of the cysteine pattern (shaded blocks) with that of one zinc finger protein (Swiss‐Prot accession number Q8R151) and one keratin‐associated protein (Swiss‐Prot accession number Q64507) of Mus musculus. Fig. S3. 3D structure prediction with AlphaFold2 of the 6 putative molluscan shell sequences that were found to be homologous to accripin11: (A) Atrina pectinata, (B) Crassostrea virginica, (C) Mytilus galloprovincialis, (D) Pinctada fucata, (E) Pinctada maxima and (F) Pinctada margaritifera. Each of these predictions shows the two antiparallel alpha helices. With the exception of the protein C, all of them exhibit a disordered C‐terminus. Fig. S4. Quantification of Accripin11 by ELISA in ASMp1 and ASMn extracts. The curve was obtained with recombinant Accripin11, serially diluted (100 to 0.7 ng) and tested with the anti‐accripin11 antibody, diluted 1000 times. (A) Calibration curve; the red line represents the linear fit and the insert table includes the linear equation and its parameters. (B) List of absorbance values obtained with ASMp1 and ASMn, both tested at concentrations ranging from 800 to 6.25 ng per well. The absorbance value of ASMp1 at 200 ng/well is almost equal to the value of accripin11 at 25 ng/well. Note that all absorbance values measured in ASMn correspond to blank values, indicating the absence of accripin11 in the extract. Fig. S5. Complete amino acid (AA) sequence of recombinant accripin11 with the StrepTag2 tag. The first two AAs (in red) belong to the pT7 expression vector. AAs in blue (from position 106 to position 115) represent the StrepTag2 tag. [file FEB4-13-10-s001.pdf]

Supplementary information

```
cgg ttt ggt ttt gtt ggc cga ccg ggc gct aga agt ccg aaa tat tga gcg ctt tgt caa
att aga ccc aaa gtg aat caa tct ttc aag atg agg ctg ctt atc gta ttc atc ctt ttc
                                        M   R   L   L   I   V   F   I   L   F
gtg acc cta gcg cag gtt ttc gcc aag ccg gcg tca aat cga cgc aac aga tat atg aac
V   T   L   A   Q   V   F   A   K   P   A   S   N   R   R   N   R   Y   M   N
atg gtt ggg agt gtc ttg gac aac tgt aga aga aag tgc atc ttc gac aat ttc tca tgt
M   V   G   S   V   L   D   N   C   R   R   K   C   I   F   D   N   F   S   C
aac atc cct tgt cga ctt ttc tac aca aca caa agg act tac aaa gat tgc gca caa caa
N   I   P   C   R   L   F   Y   T   T   Q   R   T   Y   K   D   C   A   Q   Q
tgt acc cga gat agg gaa acg tgt ttt ggt gaa tgt acg gct aac cat ggt ccc aag aaa
C   T   R   D   R   E   T   C   F   G   E   C   T   A   N   H   G   P   K   K
gct aca cct gcc cct acc acc gct gct cca aaa cca gca aag gaa ccg agt tcc gct gac
A   T   P   A   P   T   T   A   A   P   K   P   A   K   E   P   S   S   A   D
gac gat gat gac gaa tcc gat gaa tcg ttc gac tga gca gtg ttc ctg att act aag tca
D   D   D   D   E   S   D   E   S   F   D   *
aca tgt aac taa aca aaa cag ctg gta gcg gat gat agt ttt aat cac ttg tta tcg aag
tac att ttg tga act gtg tgt act cac tac gac aac gga agt tga aac aaa aca aaa ttt
tta aca cta tta atg gcc aaa gaa ttt atc ata ttt cta gat tcg ttc tat att ctt taa
aaa aaa tcc agt aat atg tca ctt gta att ata cga tga aag cac aaa ata tca aaa acg
aaa att cga aga ata ttt ttg tta aaa tta tca tat atc aag atc aga cgc aag taa gga
tga aat att tca aat caa atc acc aca att tct tag aat taa tgt tct gga aag aac aaa
aac att tca agt ctg gat aca atc agg aaa tac tat aaa caa ttt gtt ctc tgc cat ttc
ttt ttc gat ttt tct ttt taa tgt tta tct ata aat atg tgt ttt gtg cat taa ctt caa
att atg caa agc ttt aaa gac tgg aac agg tat atg aaa act caa aaa aat cac taa aca
atc aat caa tca tct gac atg ctc aaa tag ttc agt ggg agg tat caa ttt gta aat ctt
cct cta act tgc gta tgg cga agc tgt gaa ata ttt caa aat gat cac att taa taa gtt
cct ata aat aca aaa gtg ctt tga agg aaa gat cgg aag tct gag aac caa tcc tgc gca
aaa gaa act ttt gag gag gta cat tgt ggt tcc tcc gaa gag aag aca aca gtg ctt tat
ttt tca tat ata cta cac atg taa ata ctt tct tat tta tca ttc atg gca ttc agc atg
cca tgt atc taa tcc agt agt gtt aga ttt gat ttt aaa cag tat tta ctt tca gct tta
gtt gtt ttt tta ttg aaa gcc cat tat cat aac
```

Supplementary figure 1. Nucleotide sequence of the contig (>Pnobilis_R20673250) encoding accripin11. The amino acid sequence of accripin11 is shown under the nucleotide sequence in one-letter symbols (in blue): the first 18 amino acids in red represent the signal peptide. This amino acid sequence was found in the longest open reading frame of the translation 2F.

Accripin11 [*Pinna nobilis*]	aa 21	CRRKCIFDNFSCNIPCRLFYTTQRTYKDCAQQCTRDRETCFGEC	aa 21
Zinc finger protein [*Mus musculus*]	aa 1467	CQEPCTGECPPCQRTCQNRCVHSQCKKKCGELCSPCVEPCVWRC	aa 1510
Keratin associated protein [*Mus musculus*]	aa 144	CCKPCCCQSSCCKPCCSSGCGSSCCQSSCCKPCCCQSSCCKPCC	aa 187

Supplementary figure 2. Alignment of the cysteine pattern (*shaded blocks*) with that of one zinc finger protein (Swiss-Prot accession number Q8R151) and one keratin-associated protein (Swiss-Prot accession number Q64507) of *Mus musculus*.

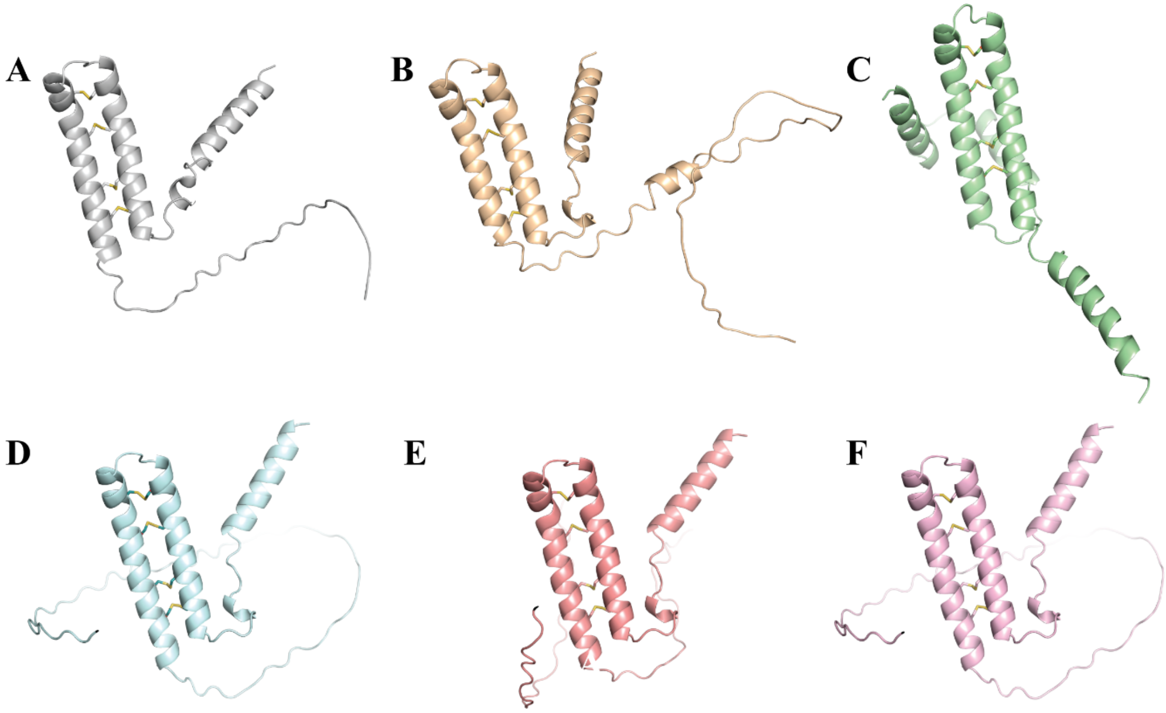

Supplementary figure 3. 3D structure prediction with AlphaFold2 of the 6 putative molluscan shell sequences that were found to be homologous to accripin11: **(A)** *Atrina pectinata* **(B)** *Crassostrea virginica* **(C)** *Mytilus galloprovincialis* **(D)** *Pinctada fucata* **(E)** *Pinctada maxima* and **(F)** *Pinctada margaritifera*. Each of these predictions shows the two antiparallel alpha helices. With the exception of the protein C, all of them exhibit a disordered C-terminus.

A

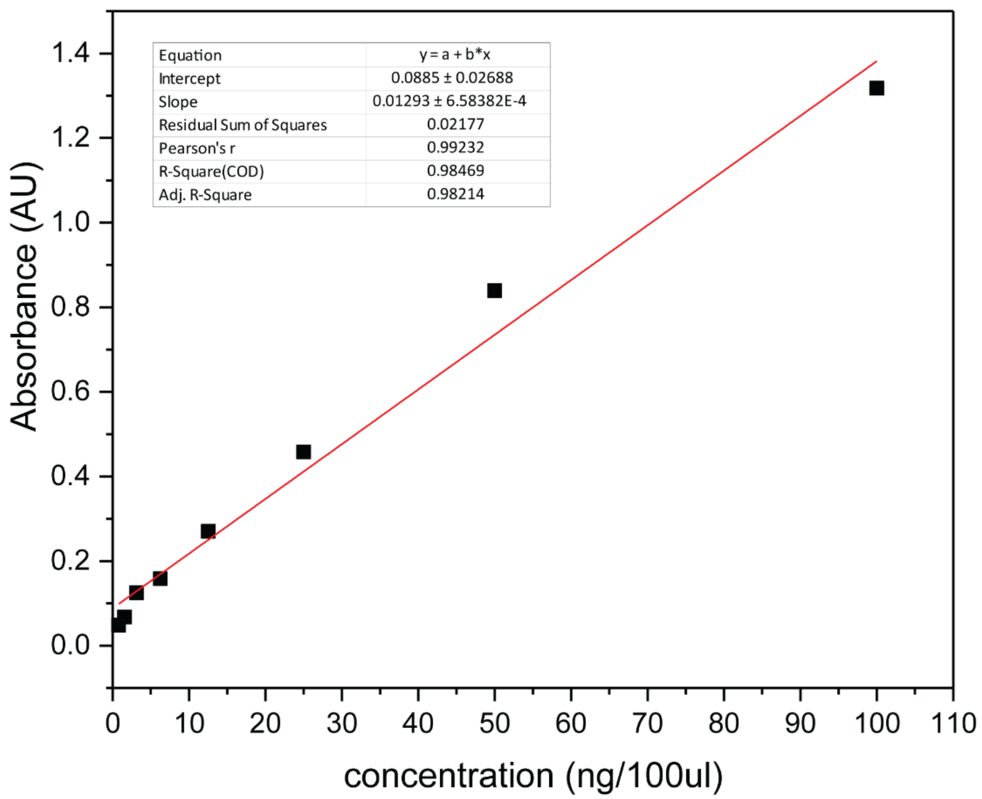

B

Concentration (ng/100 µL)		800	400	200	100	50	25	12.5	6.25
**Absorbance**	**Prism**	1.36775	0.93808	0.43508	0.24642	0.14008	0.11775	0.07308	0.01342
	**Nacre**	0.15142	0.11875	0.09908	0.10108	0.09342	0.07275	0.04942	0.04942

Supplementary figure 4. Quantification of Accripin11 by ELISA in ASM_p1 and ASM_n extracts. The curve was obtained with recombinant Accripin11, serially diluted (100 to 0.7 ng) and tested with the anti-accripin11 antibody, diluted 1000 times. A) Calibration curve; the red line represents the linear fit and the insert table includes the linear equation and its parameters. B) List of absorbance values obtained with ASM_p1 and ASM_n, both tested at concentrations ranging from 800 to 6.25 ng per well. The absorbance value of ASM_p1 at 200 ng/well is almost equal to the value of accripin11 at 25ng/well. Note that all absorbance values measured in ASM_n correspond to blank values, indicating the absence of accripin11 in the extract.

10 20 30 40 50 60 70

MGKPASNRRN RYMNMVGSVL DNCRRKCIFD NFSCNIPCRL FYTTQRTYKD CAQQCTRDRE TCFGECTANH

80 90 100 110 115

GPKKATPAPT TAAPKPAKEP SSADDDDDES DESFDSAWSH PQFEK

Supplementary figure 5. Complete amino acid (AA) sequence of recombinant accripin11 with the StrepTag2 tag. The first two AAs (in red) belong to the pT7 expression vector. AAs in blue (from position 106 to position 115) represent the StrepTag2 tag.
